# Brain-Targeted Drug Delivery Platforms for Ischemic Stroke Therapy

**DOI:** 10.34133/bmef.0055

**Published:** 2024-01-02

**Authors:** Yunhan Zhang, Haiyan Liu, Jianxun Ding

**Affiliations:** ^1^ Key Laboratory of Pathobiology Ministry of Education, Department of Anatomy, College of Basic Medical Sciences, Jilin University, Changchun 130061, P. R. China.; ^2^ Key Laboratory of Polymer Ecomaterials, Changchun Institute of Applied Chemistry, Chinese Academy of Sciences, Changchun 130022, P. R. China.; ^3^ School of Applied Chemistry and Engineering, University of Science and Technology of China, Hefei 230026, P. R. China.

Stroke is the second leading cause of death and the primary cause of permanent disability worldwide. Ischemic stroke (IS) accounts for 87% of all strokes globally and is characterized by the occlusion of cerebral vasculature due to embolic presence. Clinical treatments for IS include enzymatic thrombolysis, mechanical thrombectomy, and neuroprotection. However, these approaches have obvious limitations. First, early vascular recanalization leads to secondary cascade injuries and a high risk of hemorrhagic transformation, resulting in poor clinical outcomes for patients with IS. In addition, neuroprotective agents often fail to achieve satisfactory clinical efficacy due to inadequate drug concentrations and off-target effects [1]. Targeted stimuli-responsive nanoformulations for thrombolysis and neuroprotection have been developed to address these limitations in current clinical treatments. These nanoformulations are based on IS-specific thrombus-associated receptors and the pathological microenvironments, showing great promise in treating IS (Figure A).

**Figure.**
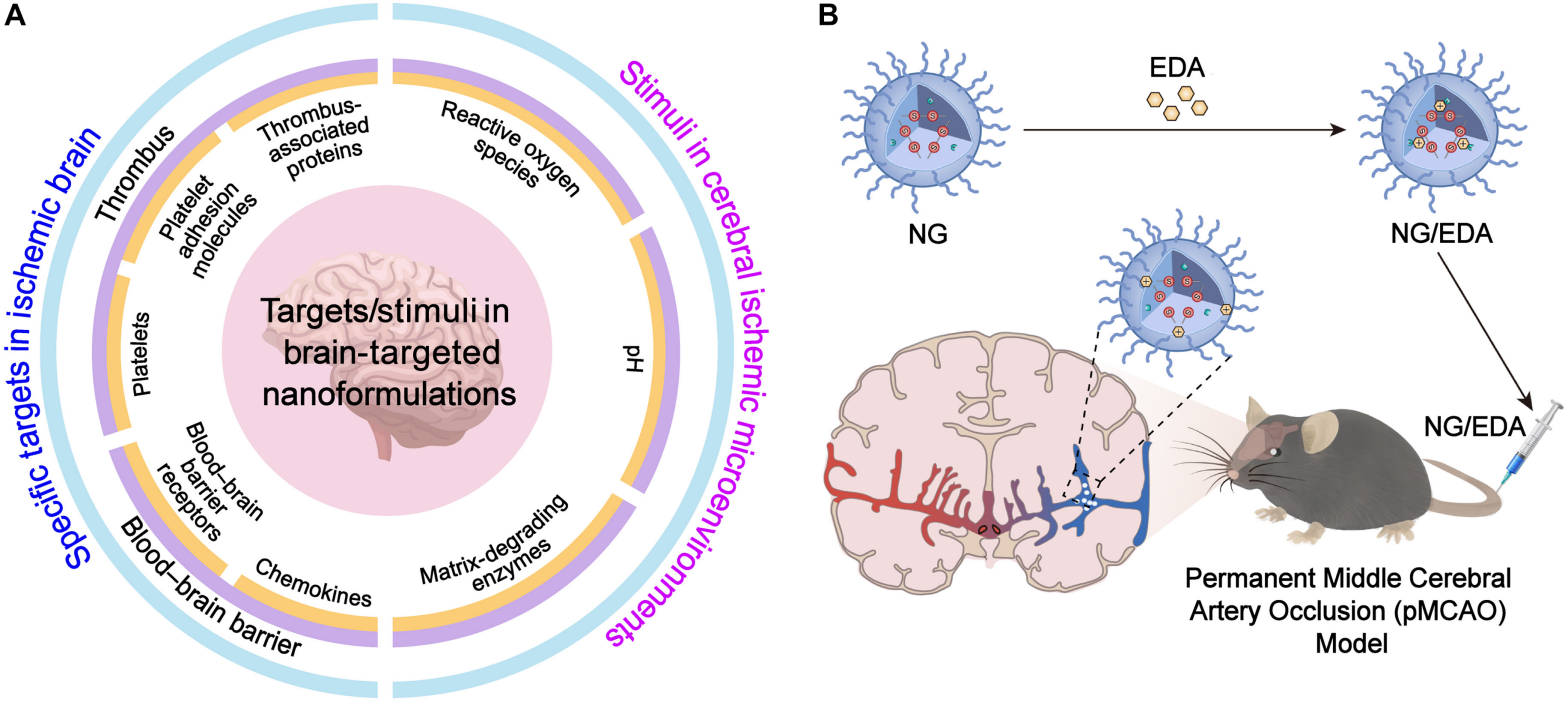
Drug delivery platforms targeting IS brain. (A) Overview of primary target categories in targeted stimuli-responsive nanoformulations. (B) Use of pH-responsive nanoformulation (NG/EDA) in IS therapy.

Immediate thrombolysis remains the primary objective in the clinical treatment of IS. Tissue-type plasminogen activator (tPA) is the sole thrombolytic drug approved by the US Food and Drug Administration. However, the short half-life and limited targeting ability of tPA restrict its effectiveness for patients with IS. Given the need for biocompatibility, nonimmunogenicity, extended circulation time, and controlled-release properties suitable for tPA delivery, the thrombus-targeted stimuli-responsive nanoformulations present a promising thrombolytic strategy for clinical application. These nanoformulations enable on-demand, precise release of thrombolytic agents at the thrombus site, leveraging high-affinity interactions with platelet adhesion molecules and thrombus-associated proteins through ligand modifications on standard nanoplatforms. This approach facilitates effective thrombolysis during the acute ischemic phase, mitigating the risk of thrombolytic-induced cerebral hemorrhage. In addition, the thrombus-targeted stimuli-responsive nanoformulations can be designed to trigger drug release at the thrombus site by exploiting elevated reactive oxygen species (ROS), overexpressed enzymes, and reduced pH within the thrombus [2]. Moreover, this method allows for the development of platforms with optimal biocompatibility and immunogenicity profiles, preserving the activity of thrombolytic drugs while preventing enzymatic degradation and rapid immune clearance. These targeted stimuli-responsive nanoplatforms thus offer a potential pathway to tailored thrombolytic therapy for patients with IS with varying degrees of embolism, promising enhanced clinical outcomes without adverse effects on drug efficacy.

The administration of neuroprotective agents has been explored as a strategy to extend the therapeutic window and mitigate secondary brain damage during IS. However, most neuroprotective agents fail in clinical trials due to several reasons: (a) Complex and highly regulated physiological barriers, such as the blood–brain barrier (BBB), severely restrict the targeted delivery of drugs to the brain; (b) inadequate blood supply to high-risk brain regions results in insufficient drug accumulation; (c) severe toxicity caused by off-target effects in patients with severe IS. An innovative drug delivery platform offers potential solutions to these challenges. Previously reported nanoformulations of neuroprotective drugs primarily relied on passive targeting mechanisms, including paracellular transport and vascular-transport-mediated transcellular pathways [3]. Recently, receptor- and adsorption-mediated transcytosis have gained prominence in the design of brain-targeted delivery platforms to enhance the active targeting of neuroprotective agents across the BBB. Following IS, receptors overexpressed in the BBB, such as transferrin receptor and intercellular adhesion molecule-1, provide specific ligands for the design of nanoplatforms [4]. The complex pathophysiological responses of IS lead to specific changes in the infarcted brain tissue, such as excessive ROS, overexpressed enzymes, and abnormal pH levels. Therefore, designing nanoformulations based on the microenvironments of diseased tissues enables effective treatment of diseases while maintaining drug activity and minimizing exposure during circulation and potential side effects in the normal tissues and organs. Building upon this approach, our team developed an edaravone-loaded pH/glutathione (reductive form) dual-responsive poly(amino acid) nanogel (NG/EDA), demonstrating its potential to deliver neuroprotective agents specifically to ischemic regions (Figure B) [5]. NG/EDA shows significant accumulation in the ischemic brain regions compared to free EDA.

Researchers have identified that heightened activity of matrix-degrading enzymes during IS leads to the breakdown of tight junctions between endothelial cells, compromising the structural integrity of the BBB. This disruption induces brain edema and impedes the effective passive targeting of therapeutic agents. Although BBB leakage may increase the accumulation of therapeutic agents in the brain, the brain uptake rates for most agents, particularly macromolecular compounds, remain insufficient for achieving effective therapeutic concentrations. The disrupted BBB facilitates the deposition of microvessel-specific proteins like vascular fibrin and triggers the expression of endothelial inflammatory factors, such as CD147, presenting a potential target for enhancing the retention of nanoparticles at the sites of vascular injury. Building upon this, the targeted delivery systems incorporating specific ligands demonstrate more efficient accumulation within ischemic brain regions. Significantly, nanoformulations designed to target inflammatory tissues based on IS-induced cellular chemotaxis—such as inflammation-induced immune cell infiltration and neural stem cell or mesenchymal stem cell migration—not only achieve efficient drug accumulation in the ischemic regions but also modulate macrophage phenotypes from inflammatory to anti-inflammatory state through targeted release of therapeutic agents.

However, pathophysiological changes following cerebral ischemia vary among patients, and the pathological characteristics differ between the initial and later stages of the condition. Therefore, selecting suitable internal stimuli and specific receptors for integration into drug delivery platforms is crucial for enhancing sustained drug accumulation and reducing neurotoxicity in developing targeted stimuli-responsive nanoformulations. Furthermore, effective treatment of IS may require multiple nanoscale strategies. For instance, the disruption of cerebral blood supply and subsequent reperfusion often triggers injury cascades, including excitotoxicity, oxidative stress, neuroinflammation, and neuronal death. Thus, developing a targeted stimuli-responsive coadministration platform for thrombolytic drugs and neuroprotective agents and leveraging microenvironment cues, such as abnormal receptors, enzymes, ROS, and pH at the ischemic site, promote nerve regeneration, repair neurological function, facilitate revascularization, and offer a promising approach for optimizing IS therapy. Another critical challenge warranting further research is that the current focus often revolves around the ability of drugs to penetrate the BBB and achieve selective release. However, preclinical studies typically rely on single animal models, which may not fully replicate human IS pathology. To address this, developing in vivo and ex vivo organ models that faithfully mimic IS injury and carefully selecting appropriate ligands and stimulus factors for nanoformulation construction significantly advance the clinical translation of brain-targeted drug delivery platforms. Given the rapid advancements and clinical applicability of nanotechnology, brain-targeted drug delivery nanoplatforms are poised to become indispensable in IS therapy.
